# Supplementary material for: Synthesis, Properties, and Application of Small-Molecule Hole-Transporting Materials Based on Acetylene-Linked Thiophene Core
Source: Molecules. 2023 Apr 26;28(9):3739. doi: 10.3390/molecules28093739 (PMC10179914; doi:10.3390/molecules28093739)
Supplement: Supplementary file 1 [file molecules-28-03739-s001.zip › molecules-2268634-supplementary.docx]

**Supporting Information**

**Synthesis, Properties, and Application of Small-Molecule Hole-Transporting Materials Based on Acetylene-Linked Thiophene Core**

Hui-Juan Yu^1,2,†^, Jing Xiao^3,4,†^, Jian Chen^3,4^, Xuefeng Ren^1,^*, Ya-E Qi^1^, Xuemei Min^1^ and Guang Shao^3,4,^*

^1^*Key Laboratory of Hexi Corridor Resources Utilization of Gansu Universities,College of Chemistry and Chemical Engineering, Hexi University, Zhangye 734000, China.*

^2^*Guangdong Key Laboratory of Animal Conservation and Resource Utilization, Guangdong Public Laboratory of Wild Animal Conservation and Utilization, Institute of Zoology, Guangdong Academy of Sciences, Guangzhou 510260, China.*

^3^*School of Chemistry, Sun Yat-sen University, Guangzhou 510006, China.*

^4^*Shenzhen Research Institute, Sun Yat-sen University, Shenzhen 518057, China.*

**Correspondence:* shaog@mail.sysu.edu.cn (G. Shao); rxfwhy@sina.com (X. Ren)

†*These authors contributed equally to this work.*

**1. General information**

Materials: All reactions were carried out under an atmosphere of argon with freshly distilled solvents, which were dried according to the standard procedures. PbI_2_ (99.99%), CH_3_NH_3_I (MAI, 99.5%), spiro-OMeTAD (99.5%), 4-*tert*-butylpyridine (*t*-BP, 96%), bis(trifluoromethane)sulfonimide lithium salt (Li-TFSI, 99.95%) were purchased from Xi’an Polymer Light Technology Corp.. All the other reagents were purchased from commercial sources and used without further purification unless otherwise noted.

Measurements: ^1^H and ^13^C NMR spectra were recorded at 25 °C on a 400 MHz nuclear magnetic resonance spectrometer (Bruker Avance III). Chemical shifts were reported relative to CDCl_3_ for ^1^H and ^13^C [^1^H NMR (CDCl_3_): *δ* =7.26 ppm, ^13^C NMR (CDCl_3_): *δ* = 77.00 ppm]. HRMS spectra were recorded on a Bruker ESI-Q-TOF maXis 4G mass spectrometer with electrospray ionization (ESI) technique. Cyclic voltammetry (CV) measurements were measured on Zahner Zennium electrochemical workstation with a three-electrode system: a platinum wire as working electrode, Ag/AgNO_3_ (0.01 M in acetonitrile) as reference electrode and a platinum sheet as counter electrode. All CV measurements were carried out in CH_2_Cl_2_ using tetrabutylammonium hexafluorophosphate (TBAPF_6_, 0.1 mol L^–1^) as the supporting electrolyte with a scan rate of 50 mV s^–1^ at room temperature, and each solution (1.0 × 10^–4^ mol L^–1^) was purged with argon for 10 min before the measurement. Spiro-OMeTAD was used as an external standard. The density functional theory (DFT) calculations were carried out by the Gaussian 09W program with the B3LYP exchange correlation functional and 6-31G basis set. The results were visualized by using GaussView program. UV–Vis absorption spectra were recorded with a SHIMADZU UV-3600 UV–VIS–NIR spectrometer. Steady state photoluminescence spectra and time-resolved photoluminescence spectra were measured on a SHIMADZU RF-5301PC or Edinburgh Instruments FLS980 spectrometer. For the time-resolved photoluminescence measurements, a pulsed laser was employed as an excitation source (a wavelength of 406.2 nm and a pulse width of 58.8 ps). Thermogravimetric analysis (TGA) was measured on a NETZSCH STA 449 F3 Jupiter thermogravimetric analyzer from 35 to 900 °C at a heating rate of 10 K/min under N_2_ atmosphere. Differential scanning calorimetry (DSC) was measured on a NETZSCH 204 F1 Phoenix differential scanning calorimeter at a heating and cooling rate of 10 K/min under N_2_ atmosphere. Water contact angle was measured on a Krüss DSA 100 drop shape analyzer. Scanning electron microscope (SEM) image was observed on a Hitachi SU8010 field-emission SEM. Atomic force microscope (AFM) image was obtained on a Bruker MultiMode8 AFM in ScanAsyst mode. The *J*–*V* curves were recorded by using a Keithley 2400 source meter under simulated AM 1.5 G (100 mW cm^–2^) illumination provided by a solar light simulator (ABET Technologies, Sun 3000). A 1000 W xenon arc lamp (Oriel, Model: 6271) was used as the light source and the incident light intensity was calibrated with an NREL standard Si solar cell. The *J*–*V* curves of all devices were measured with the active area of 0.09 cm^2^.

**2. Synthesis of HTMs**

**Scheme S1**. Synthetic routes of **CJ-05**, **CJ-06** and **CJ-07**

(1) Synthesis of compound **1**

A two-necked flask was charged with *p*-toluenesulfonic acid monohydrate (200.1 mg, 1.052 mmol, 0.10 equiv.), 3,4-dimethoxylthiophene (1515.4 mg, 10.510 mmol, 1.00 equiv.), glycol (2658.5 mg, 42.831 mmol, 4.08 equiv.) and toluene (22 mL), and was then purged with argon. The mixture was heated to 130 ℃ and kept under reflux for 48.0 hours. After the removal of solvent by rotary evaporation, the residue was purified by column chromatography with petroleum ether as eluent, giving compound **1** as a colorless oil (201.3 mg, 80.39%). ^1^H NMR (400 MHz, CDCl_3_): *δ* (TMS, ppm) = 6.32 (s, 2H), 4.19 (s, 4H), coinciding with the reported data^1^.

**Figure S1.** ^1^H NMR spectra of compound **1** (400 MHz, CDCl_3_)

(2) Synthesis of compound **2**

A round bottom flask was charge with compound **1** (490.3 mg, 3.448 mmol, 1.00 equiv.) and *N*,*N*-dimethylformamide (2 mL), and was then purged with argon. The mixture was kept at 3 °C and shaded. A solution of *N*-iodosuccinimide (1708.1 mg, 7.592 mmol, 2.20 equiv.) in *N*,*N*-dimethylformamide (12 mL) was added into the mixture under stirring. After the completion of addition, the reaction was further stirred at 3 °C for 2.0 hours and room temperature for 65.0 hours. The mixture was treated with aqueous sodium thiosulfate solution (0.1 M) till the reddish brown faded and was then extracted with ethyl acetate. The organic phase was separated, dried with anhydrous magnesium sulphate, and filtered. After the removal of solvent by rotary evaporation, the residue was purified by column chromatography with petroleum ether/ethyl acetate (50/1, v/v) as eluent, giving compound **2** as a pale yellow solid (1152.6 mg, 84.85%). ^1^H NMR (400 MHz, CDCl_3_): *δ* (TMS, ppm) = 4.26 (s, 4H), coinciding with the reported data^2^.

**Figure S2.** ^1^H NMR spectra of compound **2** (400 MHz, CDCl_3_)

(3) Synthesis of compound **3**

A two-necked flask was charged with catechol (3312.8 mg, 30.086 mmol, 2.83 equiv.), *p*-toluenesulfonic acid monohydrate (222.4 mg, 1.169 mmol, 0.11 equiv.), 3,4-dimethoxyl-thiophene (1532.4 mg, 10.628 mmol, 1.00 equiv.) and toluene (20 mL), and was then purged with argon. The mixture was heated to 130 ℃ and kept under reflux for 42.0 hours. After the removal of solvent by rotary evaporation, the residue was purified by column chromatography with petroleum ether as eluent, giving compound **3** as a white solid (743.2 mg, 36.76%). ^1^H NMR (400 MHz, CDCl_3_): *δ* (TMS, ppm) = 6.92 (s, 4H), 6.43 (s, 2H), coinciding with the reported data^3^.

**Figure S3.** ^1^H NMR spectra of compound **3** (400 MHz, CDCl_3_)

(4) Synthesis of compound **4**

A round bottom flask was charge with compound **3** (1172.4 mg, 6.163 mmol, 1.00 equiv.), *N*-iodosuccinimide (3051.4 mg, 13.563 mmol, 2.20 equiv.) and *N*,*N*-dimethylformamide (24 mL), and was then purged with argon. The mixture was shaded and stirred at room temperature for 28.0 hours. The mixture was treated with aqueous sodium thiosulfate solution (0.1 M) till the reddish brown faded and was then extracted with ethyl acetate. The organic phase was separated, dried with anhydrous magnesium sulphate, and filtered. After the removal of solvent by rotary evaporation, the residue was purified by column chromatography with petroleum ether/ethyl acetate (50/1, v/v) as eluent, giving compound **4** as a white solid (2624.4 mg, 96.34%). ^1^H NMR (400 MHz, CDCl_3_): *δ* (TMS, ppm) = 7.04–7.01 (m, 2H), 6.99–6.96 (m, 2H).

**Figure S4.** ^1^H NMR spectra of compound **4** (400 MHz, CDCl_3_)

(5) Synthesis of compound **5**

A two-necked flask was charged with aniline (474.9 mg, 5.099 mmol, 1.00 equiv.), 4-bromoanisole (2458.4 mg, 10.505 mmol, 2.06 equiv.), cuprous iodide (191.8 mg, 1.007 mmol, 0.20 equiv.), 1,10-phenathroline monohydrate (199.4 mg, 1.006 mmol, 0.20 equiv.), potassium tert-butoxide (3370.6 mg, 30.038 mmol, 5.89 equiv.) and toluene (40 mL), and was then purged with argon. The mixture was heated to 130 ℃ and kept under reflux for 39.0 hours. After the removal of solvent by rotary evaporation, the residue was purified by column chromatography with petroleum ether/ethyl acetate (50/1, v/v) as eluent, giving compound **5** as a yellow solid (946.4 mg, 60.78%). ^1^H NMR (400 MHz, CDCl_3_): *δ* (TMS, ppm) = 7.16 (t, *J* = 8.0 Hz, 2H), 7.04 (d, *J* = 8.0 Hz, 4H), 6.93 (d, *J* = 8.0 Hz, 2H), 6.85 (t, *J* = 8.0 Hz, 1H), 6.81 (d, *J* = 8.0 Hz, 4H), 3.78 (s, 6H), coinciding with the reported data^4^.

**Figure S5.** ^1^H NMR spectra of compound **5** (400 MHz, CDCl_3_)

(6) Synthesis of compound **6**

A round bottom flask was charge with compound **5** (2074.2 mg, 6.792 mmol, 1.00 equiv.), *N*-iodosuccinimide (1682.4 mg, 7.478 mmol, 1.10 equiv.) and *N*,*N*-dimethyl-formamide (16 mL), and was then purged with argon. The mixture was shaded and stirred at room temperature for 25.0 hours. The mixture was treated with aqueous sodium thiosulfate solution (0.1 M) till the reddish brown faded and was then extracted with ethyl acetate. The organic phase was separated, dried with anhydrous magnesium sulphate, and filtered. After the removal of solvent by rotary evaporation, the residue was purified by column chromatography with petroleum ether/ethyl acetate (50/1, v/v) as eluent, giving compound **6** as a yellow solid (2763.5 mg, 94.34%). ^1^H NMR (400 MHz, CDCl_3_): *δ* (TMS, ppm) = 7.40 (d, *J* = 8.0 Hz, 2H), 7.03 (d, *J* = 8.0 Hz, 4H), 6.82 (d, *J* = 8.0 Hz, 4H), 6.67 (d, *J* = 8.0 Hz, 2H), 3.78 (s, 6H), coinciding with the reported data^5^.

**Figure S6.** ^1^H NMR spectra of compound **6** (400 MHz, CDCl_3_)

(7) Synthesis of compound **7**

A round bottom flask was charge with compound **6** (2606.1 mg, 6.043 mmol, 1.00 equiv.), trimethylsilylacetylene (1.7 mL, 12.289 mmol, 2.03 equiv.), tetrakis(triphenylphosphine)-palladium (350.2 mg, 0.303 mmol, 0.05 equiv.), cuprous iodide (57.9 mg, 0.304 mmol, 0.05 equiv.), diisopropylamine (4 mL) and toluene (16 mL), and was then purged with argon. The mixture was heated to 70 °C and stirred for 24.5 hours. After the removal of solvent by rotary evaporation, the residue was purified by column chromatography with petroleum ether/ethyl acetate (50/1, v/v) as eluent, giving compound **7** as a brown oil (2288.0 mg, 94.28%). ^1^H NMR (400 MHz, CDCl_3_): *δ* (TMS, ppm) = 7.24 (d, *J* = 8.0 Hz, 2H), 7.04 (d, *J* = 8.0 Hz, 4H), 6.83 (d, *J* = 8.0 Hz, 4H), 6.79 (d, *J* = 12.0 Hz, 2H), 3.79 (s, 6H), 0.22 (s, 9H), coinciding with the reported data^6^.

**Figure S7.** ^1^H NMR spectra of compound **7** (400 MHz, CDCl_3_)

(8) Synthesis of compound **8**

A round bottom flask was charge with compound **7** (760.9 mg, 9.365 mmol, 1.00 equiv.), tetrabutylammonium fluoride trihydrate (3253.4 mg，10.312 mmol，1.10 equiv.), methanol (2 mL) and tetrahydrofuran (18 mL). The mixture was stirred at room temperature for 40.0 hours. After the removal of solvent by rotary evaporation, the residue was purified by column chromatography with petroleum ether/dichloromethane (4/1, v/v) as eluent, giving compound **8** as a yellow oil (2832.2 mg, 91.81%). ^1^H NMR (400 MHz, CDCl_3_): *δ* (TMS, ppm) = 7.26 (d, *J* = 8.0 Hz, 2H), 7.06 (d, *J* = 8.0 Hz, 4H), 6.84 (d, *J* = 8.0 Hz, 4H), 6.80 (d, *J* = 8.0 Hz, 2H), 3.80 (s, 6H), 2.99 (s, 1H), coinciding with the reported data^7^.

**Figure S8.** ^1^H NMR spectra of compound **8** (400 MHz, CDCl_3_)

(9) Synthesis of **CJ-05**

A round bottom flask was charge with compound **8** (401.5 mg, 1.219 mmol, 2.11 equiv.), 2,5-dibromothiophene (140.1 mg, 0.579 mmol, 1.00 equiv.), tetrakis(triphenylphosphine)-palladium (67.4 mg, 0.058 mmol, 0.10 equiv.), cuprous iodide (11.9 mg, 0.062 mmol, 0.11 equiv.), diisopropylamine (2 mL) and toluene (8 mL), and was then purged with argon. The mixture was heated to 85 °C and stirred for 28.0 hours. After the removal of solvent by rotary evaporation, the residue was purified by column chromatography with petroleum ether/dichloromethane (1/1, v/v) as eluent, giving **CJ-05** as a yellow solid (304.7 mg, 71.22%). ^1^H NMR (400 MHz, CDCl_3_): *δ* (TMS, ppm) = 7.28 (d, *J* = 8.0 Hz, 4H), 7.08–7.06 (m, 10H), 6.86–6.82 (m, 12H), 3.80 (s, 12H). ^13^C NMR (100 MHz, CDCl_3_): *δ* (ppm) = 156.33, 148.99, 139.94, 132.26, 131.05, 127.15, 124.57, 118.84, 114.76, 112.96, 94.64, 81.14, 55.44. HR-MS (ESI) *m*/*z*: [M]^+^ Calcd for C_48_H_38_N_2_O_4_S: 738.2547; found: 738.2564. The NMR results are coinciding with the reported data^8^.

**Figure S9.** ^1^H NMR spectra of **CJ-05** (400 MHz, CDCl_3_)

**Figure S10.** ^13^C NMR spectra of **CJ-05** (100 MHz, CDCl_3_)


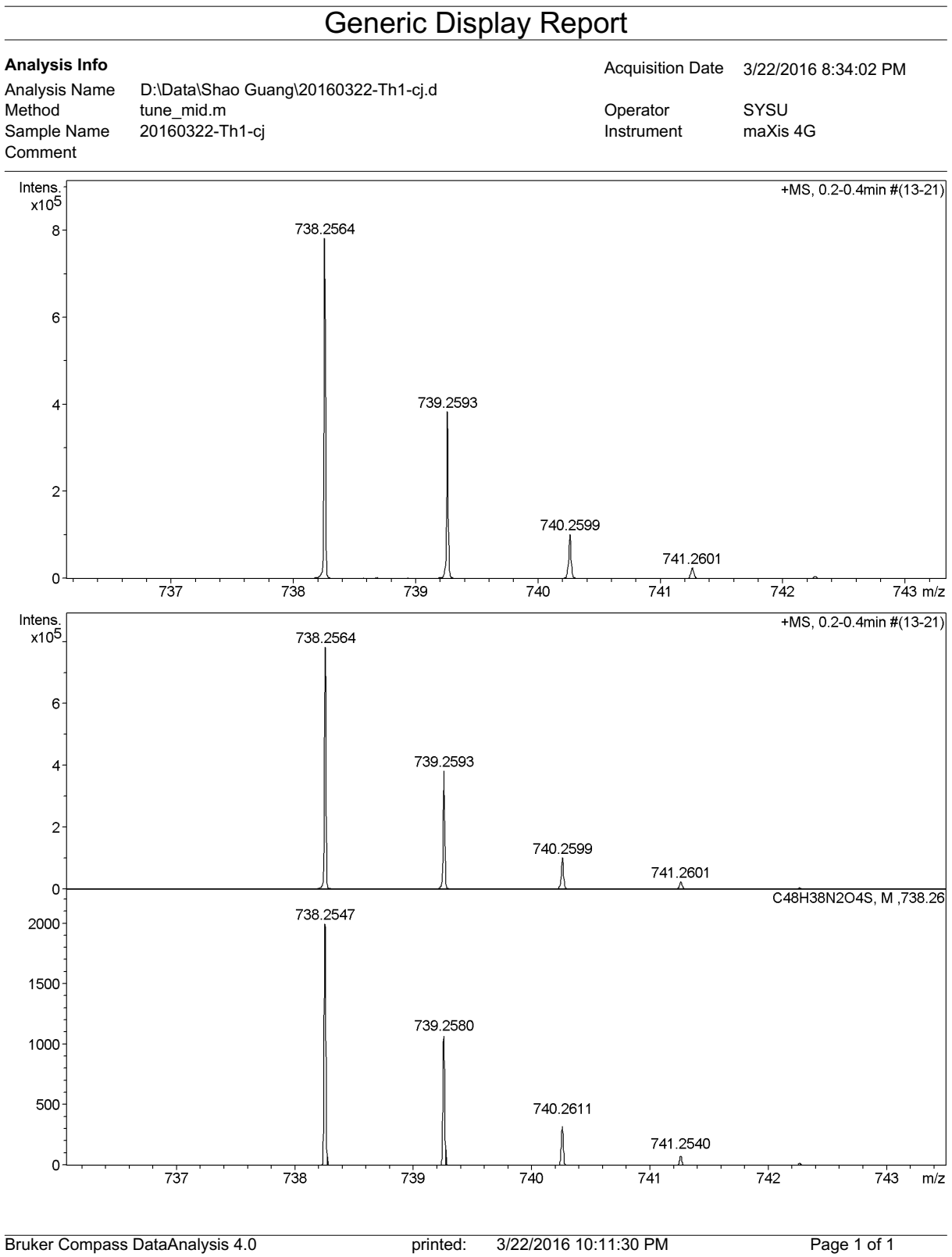


**Figure S11.** HR-MS spectra of **CJ-05**

(10) Synthesis of **CJ-06**

A round bottom flask was charge with compound **8** (1952.0 mg, 5.926 mmol, 2.10 equiv.), compound **2** (1111.3 mg, 2.821 mmol, 1.00 equiv.), tetrakis(triphenylphosphine)palladium (326.2 mg, 0.282 mmol, 0.10 equiv.), cuprous iodide (53.9 mg, 0.283 mmol, 0.10 equiv.), diisopropylamine (4 mL) and toluene (16 mL), and was then purged with argon. The mixture was heated to 85 °C and stirred for 42.5 hours. After the removal of solvent by rotary evaporation, the residue was purified by column chromatography with petroleum ether/dichloromethane (1/1, v/v) as eluent, giving **CJ-06** as a yellow solid (1313.9 mg, 58.44%). ^1^H NMR (400 MHz, CDCl_3_): *δ* (TMS, ppm) = 7.28 (d, *J* = 8.0 Hz, 4H), 7.06 (d, *J* = 8.0 Hz, 8H), 6.85–6.80 (m, 12H), 4.29 (s, 4H), 3.80 (s, 12H). ^13^C NMR (100 MHz, CDCl_3_): *δ* (ppm) = 156.27, 148.87, 142.49, 139.99, 132.32, 127.11, 118.82, 114.74, 113.17, 99.75, 97.41, 78.37, 64.76, 55.43. HR-MS (ESI) *m*/*z*: [M]^+^ Calcd for C_50_H_40_N_2_O_6_S: 796.2602; found: 796.2630. The NMR results are coinciding with the reported data^8^.

**Figure S12.** ^1^H NMR spectra of **CJ-06** (400 MHz, CDCl_3_)

**Figure S13.** ^13^C NMR spectra of **CJ-06** (100 MHz, CDCl_3_)


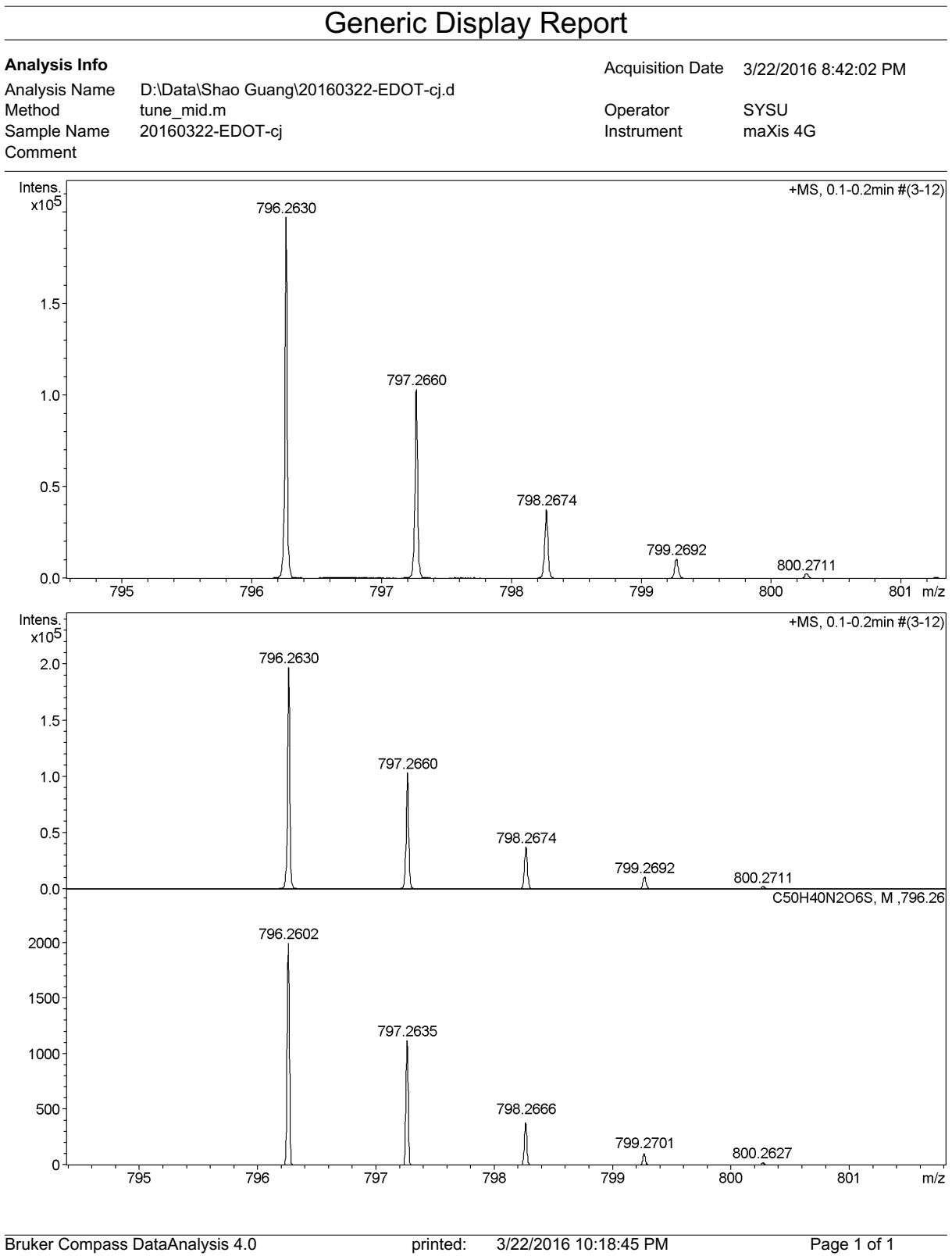


**Figure S14.** HR-MS spectra of **CJ-06**

(10) Synthesis of **CJ-07**

A round bottom flask was charge with compound **8** (1894.3 mg, 5.751 mmol, 2.10 equiv.), compound **2** (1209.1 mg, 2.735 mmol, 1.00 equiv.), tetrakis(triphenylphosphine)palladium (316.2 mg, 0.274 mmol, 0.10 equiv.), cuprous iodide (52.4 mg, 0.275 mmol, 0.10 equiv.), diisopropylamine (5 mL) and toluene (20 mL), and was then purged with argon. The mixture was heated to 85 °C and stirred for 42.5 hours. After the removal of solvent by rotary evaporation, the residue was purified by column chromatography with petroleum ether/dichloromethane/ethyl acetate (10/5/0.1, v/v/v) as eluent, giving **CJ-07** as a yellow solid (1282.8 mg, 55.51%). ^1^H NMR (400 MHz, CDCl_3_): *δ* (TMS, ppm) = 7.32 (d, *J* = 12.0 Hz, 4H), 7.07 (d, *J* = 8.0 Hz, 8H), 7.02–7.00 (m, 2H), 6.96–6.93 (m, 2H), 6.86–6.82 (m, 12H), 3.80 (s, 12H). ^13^C NMR (100 MHz, CDCl_3_): *δ* (ppm) = 156.35, 149.14, 140.22, 139.91, 139.46, 132.45, 127.17, 124.10, 118.77, 116.98, 114.77, 112.76, 100.46, 98.25, 77.48, 55.44. HR-MS (ESI) *m*/*z*: [M]^+^ Calcd for C_54_H_40_N_2_O_6_S: 844.2602; found: 844.2598.

**Figure S15.** ^1^H NMR spectra of **CJ-07** (400 MHz, CDCl_3_)

**Figure S16.** ^13^C NMR spectra of **CJ-07** (100 MHz, CDCl_3_)


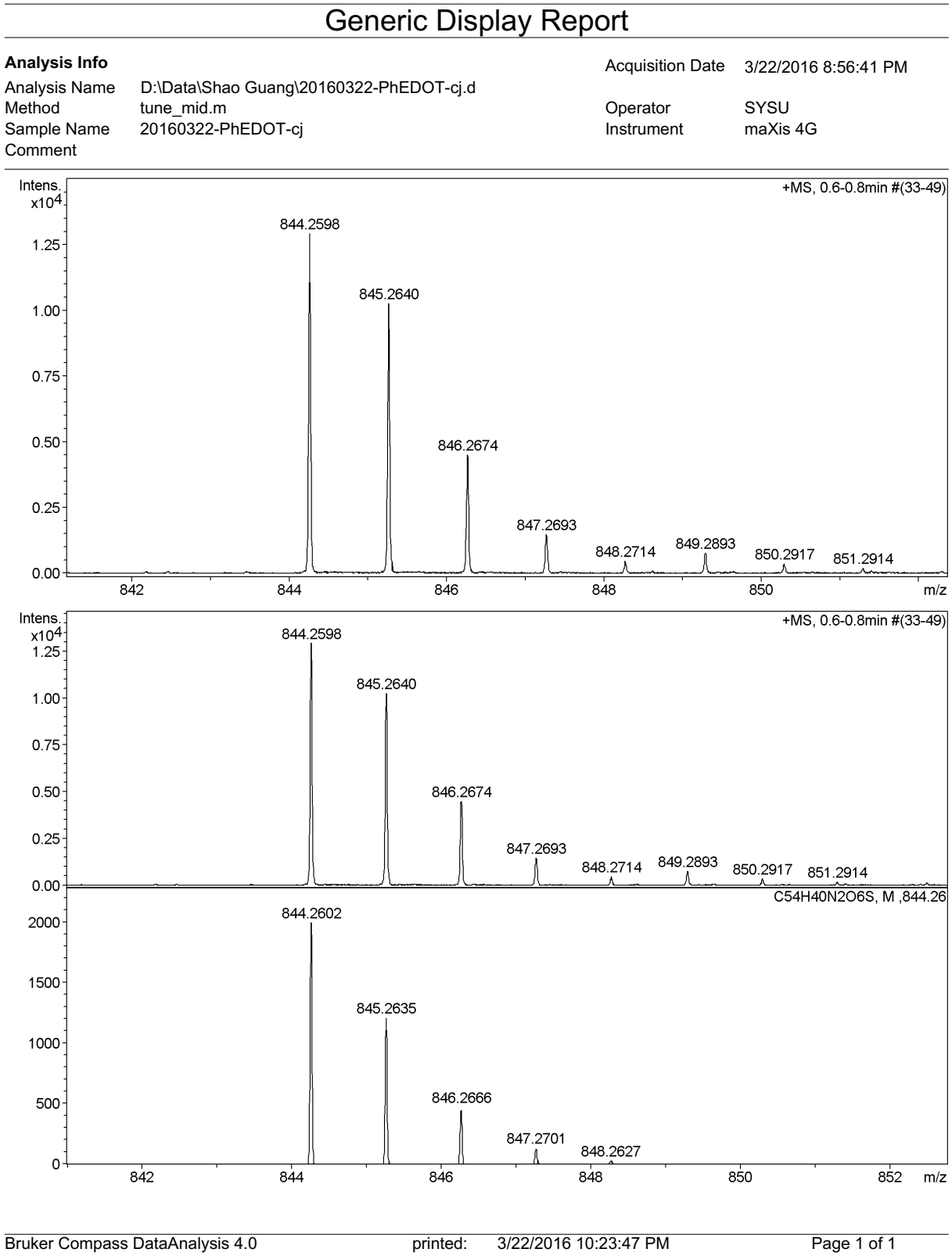


**Figure S17.** HR-MS spectra of **CJ-07**

**3. Thermal stability**

**Figure S18.** TGA of HTMs with a scan rate of 10 K/min under N_2_ atmosphere

**Figure S19.** DSC of **CJ-05** with a scan rate of 10 K/min under N_2_ atmosphere

**Figure S20.** DSC of **CJ-06** with a scan rate of 10 K/min under N_2_ atmosphere

**Figure S21.** DSC of **CJ-07** with a scan rate of 10 K/min under N_2_ atmosphere

**3. Photophysical properties**

**Figure S22.** PL spectrum of **CJ-05** solution (1.0 × 10^–5^ M in CH_2_Cl_2_,

*λ*_ex_ = 406 nm, *λ*_em_ = 526 nm; slit: 0.8 nm, 0.8 nm)

**Figure S23.** PL spectrum of **CJ-06** solution (1.0 × 10^–5^ M in CH_2_Cl_2_,

*λ*_ex_ = 407 nm, *λ*_em_ = 514 nm; slit: 0.8 nm, 0.8 nm)

**Figure S24.** PL spectrum of **CJ-07** solution (1.0 × 10^–5^ M in CH_2_Cl_2_,

*λ*_ex_ = 405 nm, *λ*_em_ = 538 nm; slit: 0.8 nm, 0.8 nm)

**Figure S25.** PL spectrum of spiro-OMeTAD solution (1.0 × 10^–5^ M in CH_2_Cl_2_,

*λ*_ex_ = 383 nm, *λ*_em_ = 424 nm; slit: 0.5 nm, 0.5 nm)

**Figure S26.** Normalized UV–Vis absorption and emission spectrum of

**CJ-05** solution (1.0 × 10^–5^ M in CH_2_Cl_2_)

**Figure S27.** Normalized UV–Vis absorption and emission spectrum of

**CJ-06** solution (1.0 × 10^–5^ M in CH_2_Cl_2_)

**Figure S28.** Normalized UV–Vis absorption and emission spectrum of

**CJ-07** solution (1.0 × 10^–5^ M in CH_2_Cl_2_)

**Figure S29.** Normalized UV–Vis absorption and emission spectrum of

spiro-OMeTAD solution (1.0 × 10^–5^ M in CH_2_Cl_2_)

**5. DFT Calculations**

**Table S1**. Optimized molecular conformations, HOMOs, and LUMOs obtained from DFT calculations

| HTM | Optimized  conformation | HOMO | LUMO |
| --- | --- | --- | --- |
| **CJ-05** | **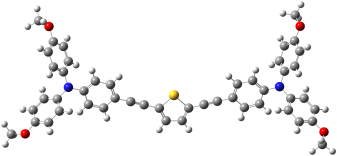**  0.52°  0.90° | **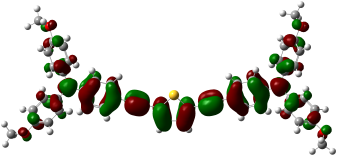** | **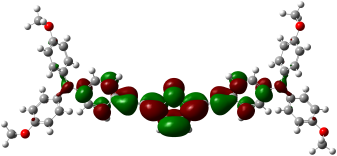** |
| **CJ-06** | **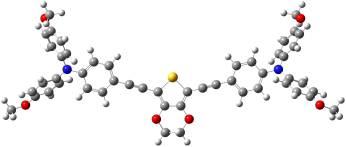**  2.83°  2.74° | **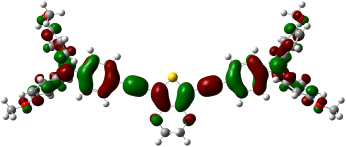** | **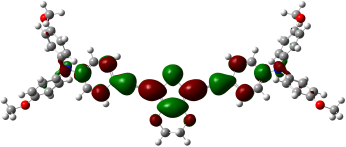** |
| **CJ-07** | **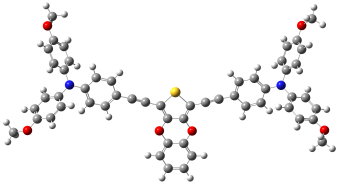**  0.35°  2.26° | **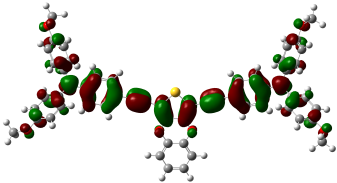** | **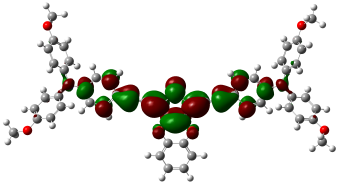** |
| spiro-OMeTAD | **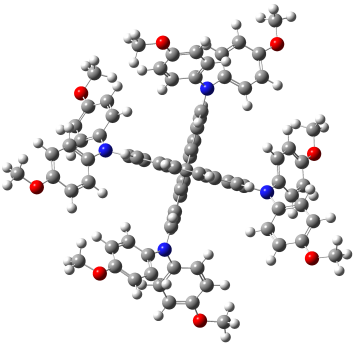** | **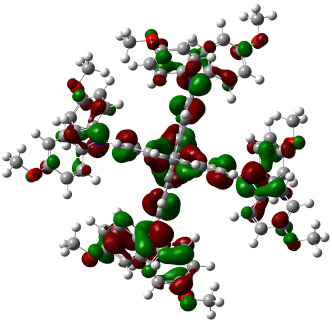** | **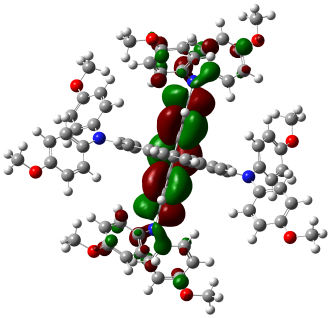** |

**Table S2.** Calculated energy levels

| HTM | HOMO (eV) | LUMO (eV) | *E*_gap_ (eV) |
| --- | --- | --- | --- |
| **CJ-05** | –4.49 | –1.63 | 2.86 |
| **CJ-06** | –4.39 | –1.58 | 2.81 |
| **CJ-07** | –4.50 | –1.68 | 2.82 |
| spiro-OMeTAD | –4.33 | –0.71 | 3.62 |

**Table S3.** Electrostatic surface potential (ESP) map and dipole moment

| HTM | Dipole  moment  (Debye, D) | ESP |  |
| --- | --- | --- | --- |
| **CJ-05** | 3.46 | 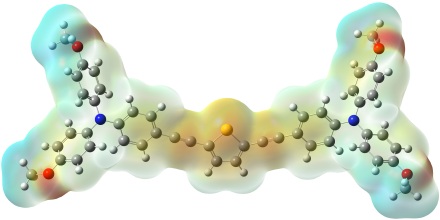 | 0.042  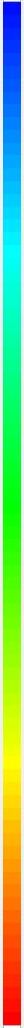  –0.042 |
| **CJ-06** | 2.36 | 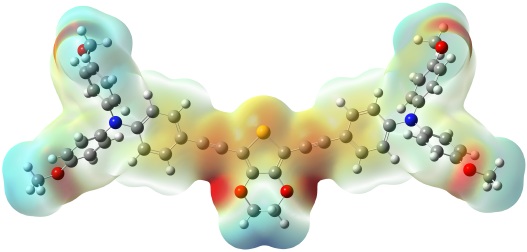 |  |
| **CJ-07** | 0.11 | 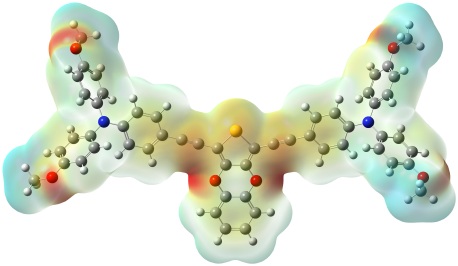 |  |
| spiro-OMeTAD | 6.19 | 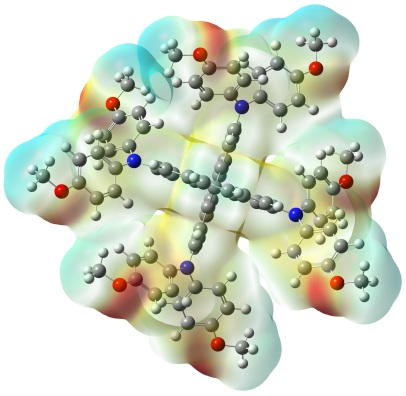 |  |

**6. Hole mobility**

Hole mobility of HTMs were measured by using a space-charge-limited current (SCLC) method. Single carrier hole-only device with a structure of ITO/PEDOT:PSS/HTM/MoO_3_/Al was fabricated to implement the measurements. Indium tin oxide (ITO) glass substrates were cleaned sequentially with detergent, deionized water, acetone, and ethanol in ultrasonic bath for 15 min, and finally cleaned with oxygen plasma for 1 min. PEDOT:PSS (40 nm) was spin-coated at 3000 r/min for 30 s and annealed at 150 °C for 20 min. The HTM layers were spin-coated at 2000 r/min for 30 s from chlorobenzene solution (20 mg/mL). MoO_3_ (10 nm) and Al (100 nm) were thermally evaporated under high vacuum sequentially onto the device. The thicknesses of HTM films were measured by a Ambios XP-1 profilometer. The current–density profiles were recorded with a Keithley 2400 sourcemeter. The SCLC characteristics in trap-free regime can be described by Mott–Gurney law:

Here, *J* is the current density, *ε*_r_ is the relative permittivity and assumed approximately to be 3, *ε*_0_ is the permittivity of the free space (8.85 × 10^–12^ F m^–1^), *μ* is the hole mobility, *V* is the effective voltage (*V* = *V*_appl_–*V*_bis_–*V*_s_, where *V*_appl_ is the applied voltage, *V*_bis_ is the built-in voltage, and *V*_s_ is the voltage drop resulting from the series resistance), and *d* is the thickness of the HTM layer.

**Figure S30**. *J*^1/2^–*V* characteristics of the hole-only devices

**7. Integrated area of steady-state photoluminescence and fitting parameters of time-resolved photoluminescence**

**Figure S31.** Integrated area of steady-state photoluminescence spectra:

(a) **CJ-05**, (b) **CJ-06**, (c) **CJ-07**, (d) spiro-OMeTAD and (e) perovskite

**Table S4.** Summary of integrated area computed from **Figure S31**

| Sample | Integrated area | Percentage (%) |
| --- | --- | --- |
| Perovskite/**CJ-05** | 4.83 × 10^6^ | 8.12 |
| Perovskite/**CJ-06** | 5.26 × 10^6^ | 9.45 |
| Perovskite/**CJ-07** | 6.12 × 10^6^ | 10.27 |
| Perovskite/spiro-OMeTAD | 2.82 × 10^6^ | 4.73 |
| Perovskite | 5.95 × 10^7^ | 100.00 |

**Table S5.** Bi-exponential fitting parameters of time-resolved photoluminescence measurements

| Sample | *τ*_1_ (ns) | frac. 1 | *τ*_2_ (ns) | frac. 2 | ave. (ns) |
| --- | --- | --- | --- | --- | --- |
| MAPbI_3_ | 7.66 | 17.51% | 145.18 | 82.49% | 121.10 |
| MAPbI_3_**/CJ-05** | 3.89 | 41.91% | 28.47 | 58.09% | 18.17 |
| MAPbI_3_**/CJ-06** | 4.70 | 42.89% | 30.67 | 57.11% | 19.53 |
| MAPbI_3_**/CJ-07** | 4.57 | 34.49% | 28.65 | 65.51% | 20.34 |
| MAPbI_3_/spiro-OMeTAD | 5.02 | 58.26% | 33.40 | 41.74% | 16.87 |

**8. Water contact angles**


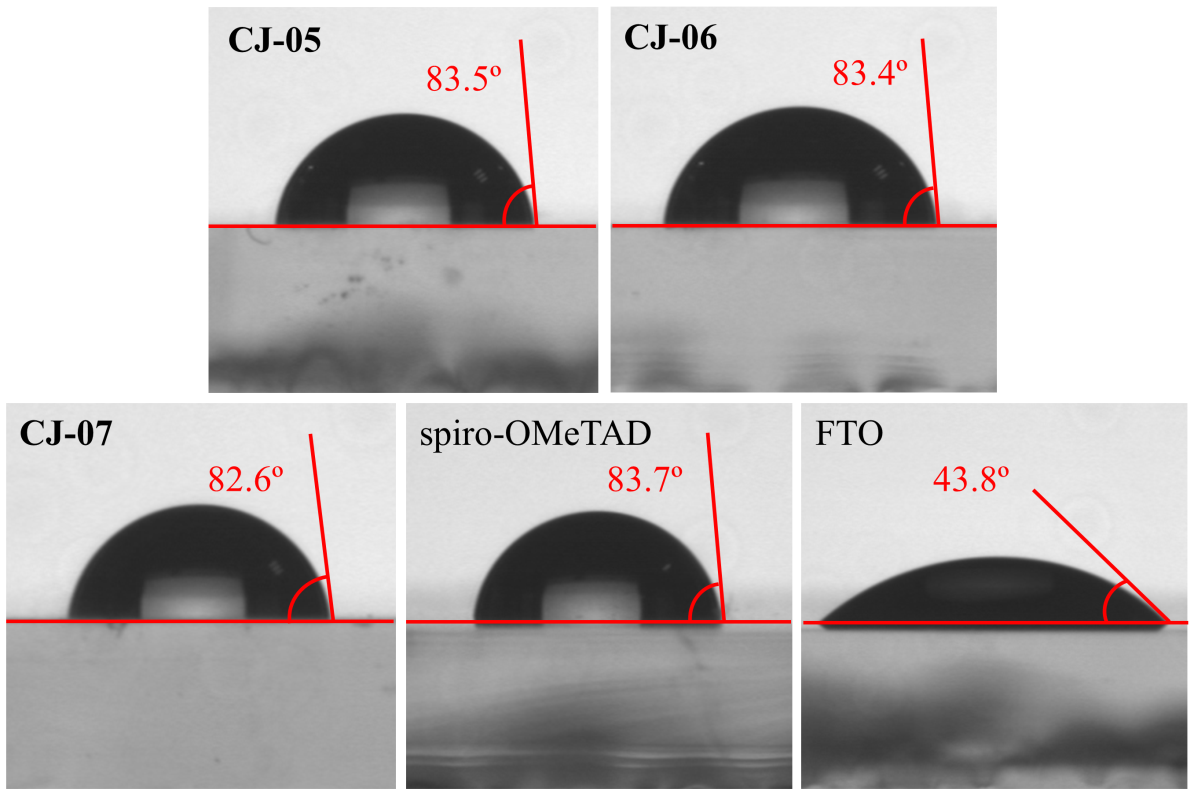


**Figure S32.** Water contact angles of pristine HTM films on the FTO substrate

**9. Surface morphologies**





**Figure S33.** SEM top-view image of perovskite film

|  | Low magnification | High magnification |
| --- | --- | --- |
| (a) |  |  |
| (b) |  |  |
| (c) |  |  |
| (d) |  | 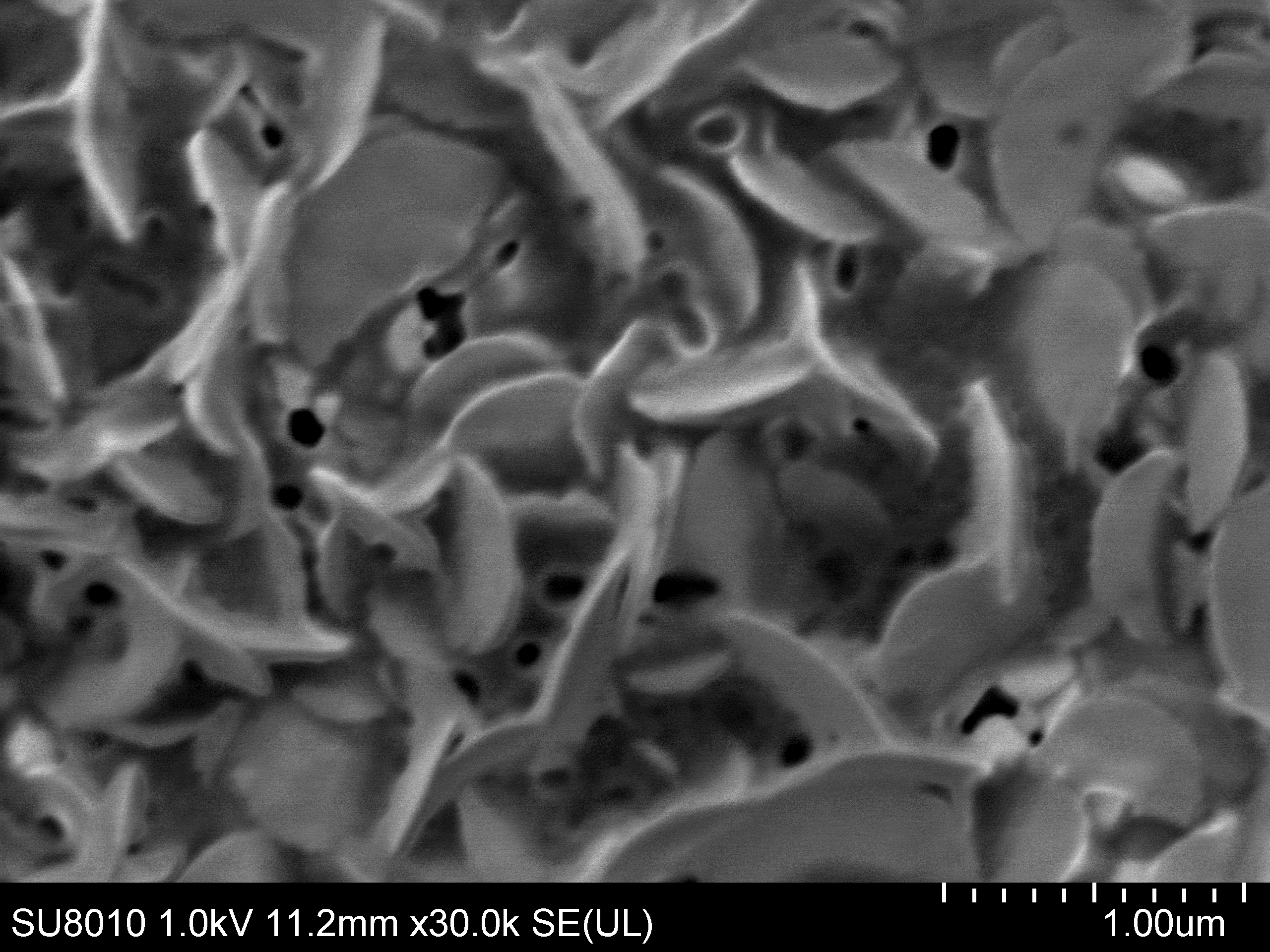 |

**Figure S34.** SEM top-view images of different HTMs coated on perovskite film:

(a) **CJ-05,** (b) **CJ-06**, (c) **CJ-07**, and (d) spiro-OMeTAD

| (a) | 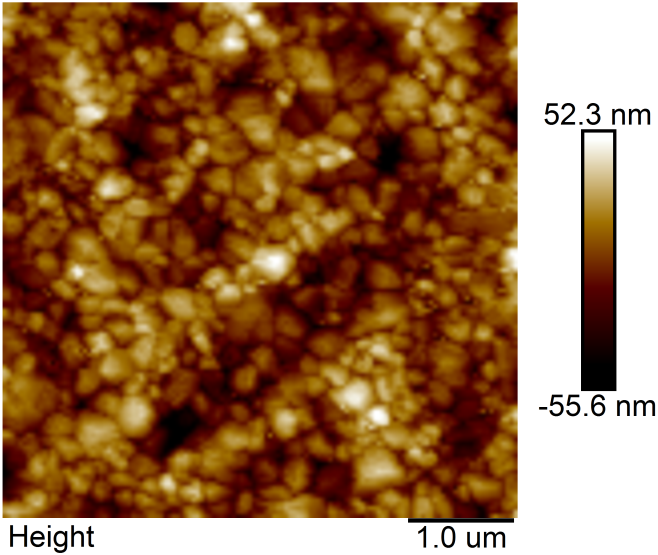  RMS = 15.4 nm | 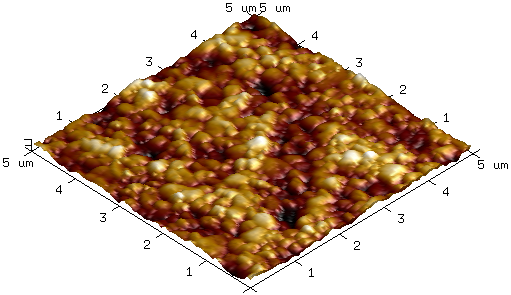 |
| --- | --- | --- |
| (b) | 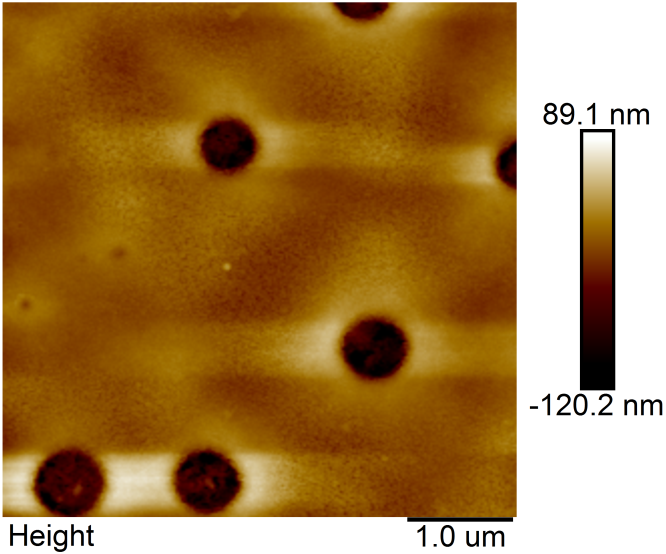  RMS = 21.0 nm | 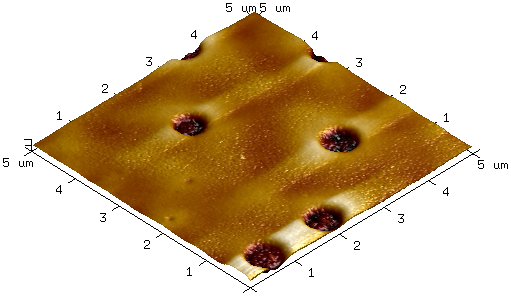   |
| (c) | 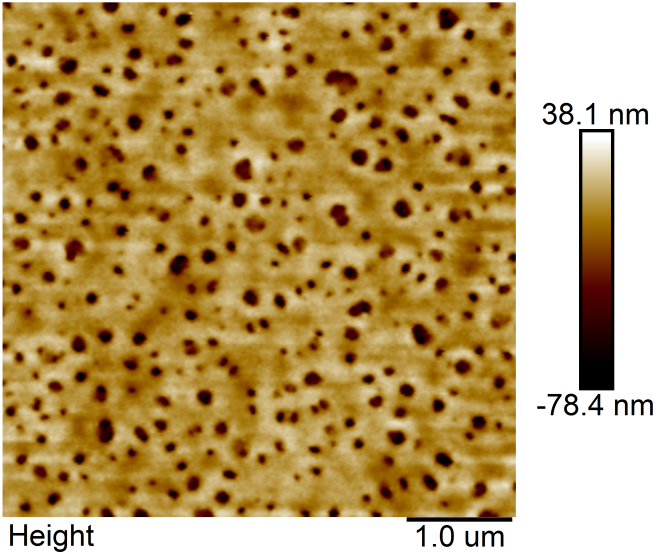  RMS = 14.7 nm | 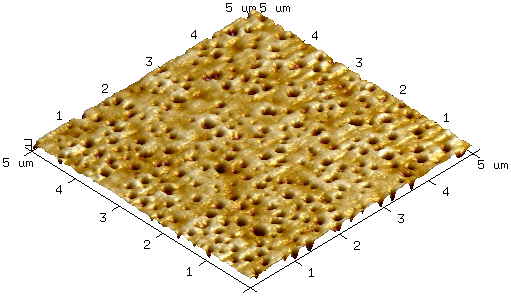   |
| (d) | 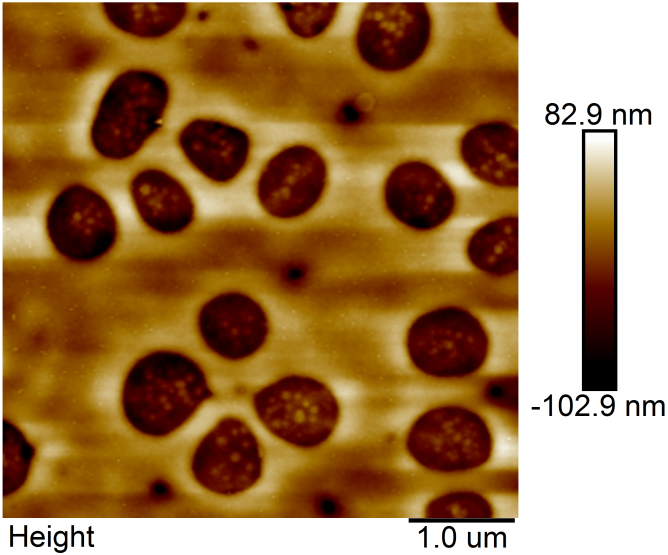  RMS = 29.3 nm | 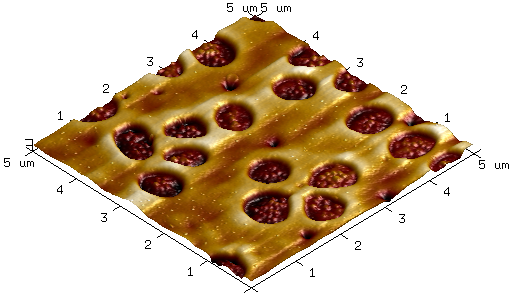   |
| (e) | 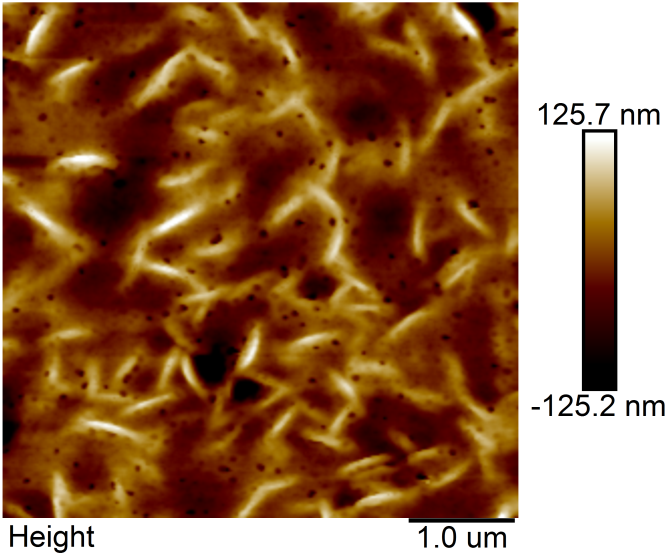  RMS = 36.2 nm | 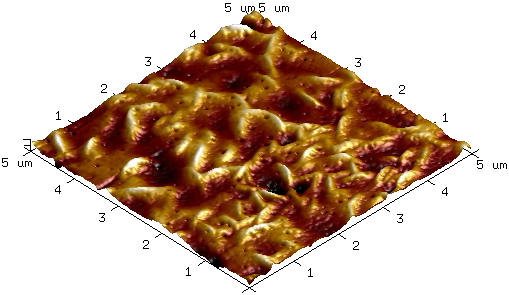   |

**Figure S35.** AFM height and corresponding 3D images of perovskite film with different layers: (a) perovskite, (b) perovskite/**CJ-05**, (c) perovskite/**CJ-06**, (d) perovskite **CJ-07**, and (e) perovskite/spiro-OMeTAD

**10. Fabrication, measurements, and photovoltaic parameters**

**Table S6.** Preparation of HTM solutions

| HTM | Concentration  (mg mL^–1^) | Li-TFSI  (mg) | FK209 (15 mol %)  (mg) | *t*-BP  (μL) | Chlorobenzene  (mL) |
| --- | --- | --- | --- | --- | --- |
| **CJ-05** | 40.0 | 9.1 | 12.2 | 28.8 | 1 |
| **CJ-06** | 50.0 | 9.1 | 14.1 | 28.8 | 1 |
| **CJ-07** | 50.0 | 9.1 | 13.3 | 28.8 | 1 |
| spiro-OMeTAD | 72.3 | 9.1 | 13.3 | 28.8 | 1 |

Fluorine-doped tin oxide (FTO) substrates were sequentially cleaned by ultrasonic bath with a solution of detergent diluted in deionized water, alkaline ethanol solution, ethanol, acetone and deionized water, each cleaning step lasted for 20 min and then dried with a nitrogen stream. The substrates were treated with UV/ozone for 30 min to remove the trace amount of organic residues. The compact TiO_2_ layer was coated on the FTO substrates by spin-coating a mildly acidic titanium (IV) isopropoxide solution at 3000 r/min for 50 s, followed by heating at 150 °C for 15 min, and then the compact TiO_2_ films were gradually heated to 500 °C and baked for 30 min. After cooling to room temperature, mesoporous TiO_2_ layer was deposited on compact TiO_2_ layer by spin-coating TiO_2_ paste (diluted in ethanol in a mass ratio of 1/7) at 5000 r/min for 30 s. The layers were then dried at 125 °C for 10 min and sintered at 500 °C for 30 min. After that, the TiO_2_ layers were treated with UV/ozone for 10 min and transferred to glove box immediately. The perovskite precursor solution was spin-coated on the mesoporous TiO_2_ layers at 3000 r/min for 55 s. During the spin-coating, chlorobenzene (100 μL) was dropped at the center of the substrates after 8 s. The precursor films were annealing at 100 °C for 20 min to form perovskite films. Subsequently, HTMs were deposited on the MAPbI_3_ films by spin-coating chlorobenzene solution at 5000 r/min for 30 s. Finally, Au electrode was thermally evaporated on the HTM under vacuum, and the active area of each device was 0.09 cm^2^.


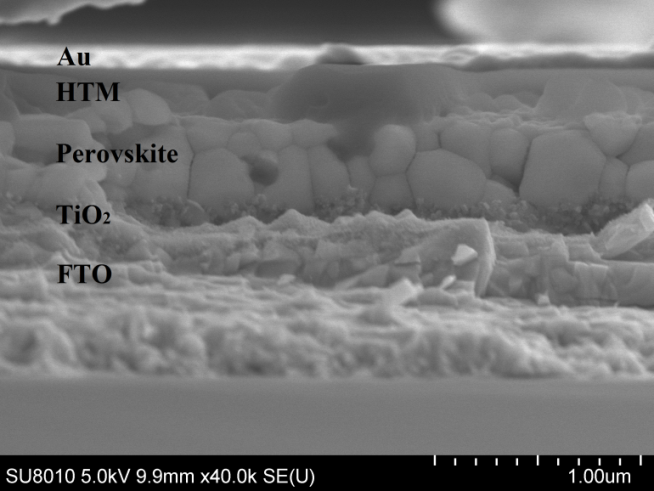


**Figure S36.** Cross-section SEM image of PSC based on **CJ-07**

**REFERENCES**

1. A. R. Agrawal, N. R. Kumar, S. Debnath, S. Das, C. Kumar, S. S. Zade. Radical-cascade avenue for 3,4-fused-ring-substituted thiophenes. *Org*. *Lett*., **2018**, *20*, 4728–4731.

2. L. Ji, R. M. Edkins, L. J. Sewell, A. Beeby, A. S. Batsanov, K. Fucke, M. Drafz, J. A. K. Howard, O. Moutounet, F. Ibersiene, A. Boucekkine, E. Furet, Z.-Q. Liu, J.-F. Halet, C. Katan, T. B. Marder. Experimental and theoretical studies of quadrupolar oligothiophene-cored chromophores containing dimesitylboryl moieties as *π*-accepting end-groups: Syntheses, structures, fluorescence, and one- and two-photon absorption. *Chem*. *Eur*. *J*., **2014**, *20*, 13618–13635.

3. T. Darmanin, J.-P. Laugier, F. Orange, F. Guittard. Influence of the monomer structure and electrochemical parameters on the formation of nanotubes with parahydrophobic properties (high water adhesion) by a templateless electropolymerization process. *J*. *Colloid* *Interface* *Sci*., **2016**, *466*, 413–424.

4. K. Rakstys, A. Abate, M. I. Dar, P. Gao, V. Jankauskas, G. Jacopin, E. Kamarauskas, S. Kazim, S. Ahmad, M. Grätzel, M. K. Nazeeruddin. Triazatruxene-based hole transporting materials for highly efficient perovskite solar cells. *J*. *Am*. *Chem*. *Soc*., **2015**, *137*, 16172–16178.

5. J.-L. Song, P. Amaladass, S.-H. Wen, K. K. Pasunooti, A. Li, Y.-L. Yu, X. Wang, W.-Q. Deng, X.-W. Liu. Aryl/hetero-arylethyne bridged dyes: The effect of planar *π*-bridge on the performance of dye-sensitized solar cells. *New* *J*. *Chem*., **2011**, *35*, 127–136.

6. T.-L. Zhou, R. T. Anderson, H.-S. Li, J. Bell, Y.-A. Yang, B. P. Gorman, S. Pylypenko, M. T. Lusk, A. Sellinger. Bandgap tuning of silicon quantum dots by surface functionalization with conjugated organic groups. *Nano* *Lett*., **2015**, *15*, 3657–3663.

7. Y. Yamaguchi, T. Ochi, Y. Matsubara, Z.-I. Yoshida. Highly emissive whole rainbow fluorophores consisting of 1,4-bis(2-phenylethynyl)benzene core skeleton: Design, synthesis, and light-emitting characteristics. *J*. *Phys*. *Chem*. A, **2015**, *119*, 8630–8642.

8. G. Nöll, M. Avola, M. Lynch, J. Daub. Comparison of alternant and nonalternant aromatic bridge systems with respect to their ET-properties. *J*. *Phys*. *Chem*. *C*, **2007**, *111*, 3197–3204.
